# The Importance of Surgical Cutting Time as a Key Performance Indicator Alongside Touchtime Utilisation in Operating Theatre Efficiency Optimisation

**DOI:** 10.7759/cureus.69660

**Published:** 2024-09-18

**Authors:** William Kirk, Reece Barter, Parisah Seyed-Safi, Andrew Davies, Sanjeeve Sabharwal

**Affiliations:** 1 Trauma and Orthopaedics, Imperial College Healthcare NHS Trust, London, GBR; 2 Orthopaedic Surgery, Imperial College London, London, GBR; 3 Trauma and Orthopaedics, Imperial College London, London, GBR

**Keywords:** getting it right first time, surgical cutting time, theatre efficiency, theatre productivity, touchtime utilisation

## Abstract

Aims

The Getting It Right First Time (GIRFT) programme has set targets to achieve 85% touchtime utilisation by 2024/25. Touchtime utilisation is a measure of theatre productivity, defined as the time from the start of anaesthesia to the time a patient leaves the theatre for all cases on a defined theatre list as a percentage of total available theatre time. No published evidence examines touchtime utilisation as a measure of theatre efficiency and its correlation with surgical cutting time. This study aims to determine if there is a statistical relationship between touchtime utilisation and surgical cutting time, and whether the use of touchtime utilisation is sufficient to inform about surgical productivity.

Methods

A retrospective analysis of two orthopaedic theatres spanning 100 days at both a major trauma centre and an elective hospital in London was performed. Electronic records identified anaesthetic start time, knife-to-skin time, end-of-procedure time, and patient-leaving-theatre time. Time intervals were calculated and the relationship between touchtime utilisation and surgical cutting time was assessed using Pearson’s correlation coefficient (r).

Results

The mean total touchtime was 403 minutes (SD, 84 minutes) at the major trauma centre and 383 minutes (SD, 103 minutes) at the elective hospital. The mean total surgical cutting time was 259 minutes (SD, 72 minutes) at the major trauma centre and 233 minutes (SD, 75 minutes) at the elective hospital, from a total available time of 510 minutes per list. There was a significant correlation between touchtime and surgical cutting time at both hospitals (elective hospital: r (198) = 0.815, p < 0.001; major trauma centre: r (198) = 0.892, p < 0.001). The mean touchtime utilisation was 79.31% for the major trauma centre and 75.20% for the elective hospital; however, the mean total surgical cutting time was 51% and 46%, respectively, of total available time.

Conclusion

Despite a good correlation between touchtime and surgical cutting time, the range between these measures suggests that using touchtime utilisation alone to measure theatre efficiency may not sufficiently inform about efficient practices. We suggest complementing touchtime utilisation data with surgical cutting time may provide more information to contextualise efficiencies or inefficiencies in an operating theatre.

## Introduction

The Getting It Right First Time's (GIRFT) national high volume low complexity (HVLC) programme has set targets to achieve 85% theatre touchtime utilisation by 2024/25. This is to support NHS England’s 2022/2023 elective surgery recovery plan post COVID-19 pandemic [1]. Touchtime is a lean concept for measuring system efficiency [2]. It identifies in a production cycle how much time is spent working on the product as a proportion of total production time [3]. In surgery, touchtime utilisation is total touchtime as a proportion of total available operating time [4]. There is a sparsity of literature with regard to the applicability of touchtime utilisation to surgical efficiency and this has not yet been shown to be a valid indicator of theatre productivity. Touchtime utilisation can be capped or uncapped. Capped theatre utilisation refers to total touchtime being calculated only within the planned session time. Uncapped touchtime utilisation refers to touchtime being calculated from the total amount of time the surgical team was operating for, including, for example, session overruns [4]. GIRFT is using capped touchtime utilisation as one of its key performance indicators.

Touchtime records from the anaesthetic start time to the time the patient leaves the theatre (combining anaesthetic time, surgical setup time, surgical cutting time, and time to leave theatre) [4]. Given this is a combined metric, it may not be a true representation of pure surgical productivity with implications for NHS waiting time directives. There are no current recommendations for a minimum percentage of touchtime that should be surgical cutting time. Literature on the topic is again sparse but one study of orthopaedic theatre utilisation demonstrated that 74% of available theatre time is used for operating [5]. Furthermore, there is no consensus on an optimal ratio of anaesthetic time to surgical cutting time but a multicentre study from Holland suggested that for more accurate scheduling purposes, surgeon-controlled time should be increased 1.33-fold to give a more accurate combined surgical and anaesthetic time [6]. This again results in surgical cutting time accounting for 75.2% of touchtime (1/1.33).

The aims of our study are to establish whether there is a relationship between touchtime utilisation and surgical cutting time and whether the use of touchtime in isolation is sufficient to inform about surgical productivity.

## Materials and methods

A total of 100 consecutive weekdays of operating lists were analysed from two large teaching hospitals at a North-West London NHS Trust. Hospitals included were a major trauma centre (MTC) and an elective hospital (EH). Each hospital had two orthopaedic theatre lists running each weekday, leading to an analysis of 200 theatre lists per site. For the EH, the period of data collection spanned from 28/09/22 to 19/06/2023. For the MTC, the period spanned from 23/09/2022 to 05/04/2023. Exclusion criteria consisted of industrial action days, weekends, and bank holiday theatre lists. These were excluded as surgical capacity on these days is often considerably disrupted when compared to normal weekday operating.

For each surgical case that was performed, the following times were recorded from the electronic health records system (Cerner™, Kansas City, MO). Anaesthetic start time, knife-to-skin time, end of procedure time and patient leaving theatre time. The total available operating time at both hospitals was eight hours and 30 minutes (510 minutes, 08:30 to 17:00 hours) (Table 1).

**Table 1:** Table summarising calculations and abbreviations used in this study.

Descriptor	Abbreviation	Definition
Touchtime	TT	Anaesthetic start time to time when the patient leaves the theatre
Total touchtime	TTT	Total of touchtime on the defined theatre list
Surgical cutting time	SCT	Knife to skin time to surgical end time
Total surgical cutting time	TSCT	Total of surgical cutting time of all cases on theatre list
Touchtime utilisation (capped)	TTU	Total touchtime as a percentage of total available time on a specified operating list
Surgical cutting time utilisation	SCTU	Surgical cutting time as a percentage of total available time on a specified operating list

The following were calculated from our data: touchtime, total touchtime, surgical cutting time, total surgical cutting time, touchtime utilisation, and surgical cutting time utilisation (Table 1).

Touchtime was measured from the anaesthetic start time to the patient out of theatre time. Total touchtime was calculated as the combined touchtime for each patient on a defined theatre list. Touchtime utilisation is calculated as total touchtime as a percentage of total available time on a defined theatre list. Surgical cutting time was measured from knife to skin time to surgical end time. Total surgical time was calculated by combining the surgical cutting times for each patient on a defined theatre list. Surgical cutting time utilisation is calculated as total surgical cutting time as a percentage of total available time on a defined theatre list. Calculations were conducted to determine the average percentage of touchtime dedicated to surgical cutting (total surgical cutting time/total touch time).

For lists that overran past 17:00, the surgical end and time out of theatre were recorded at 17:00, such that utilisation time was not artificially inflated compared to total available time, in keeping with capped touchtime utilisation.

Statistics

Continuous variables are presented as mean (SD). Durations are reported as the total time in minutes. A two-sample t-test was used to compare mean durations between the EH and MTC. The relationship between touchtime and surgical cutting time was assessed using Pearson’s correlation coefficient (r). A threshold of P < 0.05 was considered significant. Analyses were performed using Stata SE version 16 (StataCorp LLC, College Station, TX).

## Results

The average number of cases completed over a 510-minute (eight hours and 30 minutes) operating list was 2.0 (SD, 0.14) at the MTC and 2.72 (SD, 0.90) at the EH. On average, more cases were completed at the EH per day, but the average total touchtime (383 minutes vs. 403 minutes, p = 0.04), as well as the average total surgical cutting time (233 minutes vs. 259 minutes, p = 0.001), was lower. At the MTC, there was an average surgical cutting time utilisation of 51% and at the EH, it was 46%.

The touchtime utilisation for the EH and MTC both fell below the target of 85% touchtime utilisation as laid out by GIRFT (75% at the EH and 79% at the MTC) (Table 2).

**Table 2:** Table comparing results at the elective hospital and the major trauma centre (MTC).

	Elective hospital	MTC
Mean number of cases performed	2.0 (SD, 0.14)	2.71 (SD, 0.90)
Mean total touchtime (minutes)	383 (SD, 103)	383 (SD, 84)
Mean total surgical cutting time (minutes)	233 (SD, 75)	259 (SD, 72)
Mean total touchtime not cutting (minutes)	149 (SD, 49)	145 (SD, 46)
Mean empty theatre time (minutes)	126 (SD, 103)	105 (SD, 83)
Mean touchtime utilisation (%)	75%	79%
Mean surgical cutting time utilisation (%)	46%	51%
Mean total surgical cutting time as a percentage of total touchtime (%)	60%	63%

At the MTC, for lists that met or exceeded the targeted 85% touchtime utilisation (85/200 lists), there was an average total surgical cutting time of 303 minutes (SD, 51 minutes), compared with 227 minutes (SD, 62 minutes) on lists that did not meet 85% touchtime utilisation.

At the EH, for lists that met or exceeded the targeted 85% touchtime utilisation (81/200 lists), there was an average total surgical cutting time of 297 minutes (SD, 38 minutes) compared to 191 minutes (SD, 64 minutes) on lists that did not meet 85% touchtime utilisation.

Although the average total surgical cutting time was higher on the theatre lists with a higher total touchtime (at or above the targeted 85%), the average proportion of total touchtime consisting of surgical cutting was 64.75% for the MTC and 62.64% for the EH (Table 3).

**Table 3:** Comparison of operating lists with, greater than and equal to, or less than 85% touchtime utilisation. MTC: major trauma centre.

	Elective hospital ≥85% touchtime utilisation	Elective hospital <85% touchtime utilisation	MTC ≥85% touchtime utilisation	MTC <85% touchtime utilisation
Number of lists	81	119	85	115
Average total surgical cutting time (minutes)	297	191	303	227
Average proportion of touchtime used for surgical cutting time (%)	63%	59%	65%	63%
Number of lists with ≥75% surgical cutting time of touchtime (%)	6	6	13	9

Using Pearson’s coefficient, there was a strong positive correlation between total touchtime and total surgical cutting time at both the MTC and EH (MTC: r (198) = 0.815, p < 0.001; EH: r (198) = 0.892, p < 0.001).

At the EH, the average empty theatre time was 126 minutes (SD, 103 minutes). At the MTC, the average was 105 minutes (SD, 83 minutes) (p < 0.001).

## Discussion

Comparatively, over the period of data collection, the MTC and the EH had a similar performance using GIRFT’s measure of operating theatre efficiency. Touchtime utilisation correlated well with surgical cutting time; however, the results of this observational study demonstrate that the discrepancy between theatre utilisation and operating time highlights the weakness of measuring efficiency with touchtime utilisation alone. Although the average total surgical cutting time was higher on the theatre lists with a higher touchtime utilisation (at or above the target 85%), the average proportion of touchtime used for surgical cutting was 64.75% for the MTC and 62.64% for the EH. In both hospitals, on average, less than two-thirds of the time a patient spent in theatre was surgical cutting time.

Of the 166 theatre lists across both hospitals with 85% or greater touchtime utilisation, only 19 (11.44%) had a total surgical cutting time as a proportion of total touchtime greater than or equal to 75%. Touchtime utilisation in isolation can erroneously provide the illusion that surgical productivity is optimised. An example of this would be a situation where either anaesthetic time was extended to one hour due to delays in the anaesthetic room, or if the patient remained in the operating theatre post-surgery for an additional hour due to lack of a recovery bed. While the surgery itself may only have taken 45 minutes. Touchtime utilisation would not pinpoint the causes of these inefficiencies.

For a more detailed analysis of surgical efficiency, we propose a target of 75% of touchtime to be used as surgical cutting time, based on a range of 74-75.2% from the rudimentary literature available [5,6]. High-intensity theatre lists trialled in specific hospitals can result in 90% surgical cutting time over the course of an operating list, so this suggested target of 75% of touchtime should be a minimum set standard [7]. It is important that such measures are further validated based on surgical specialties, and standardised against case complexity as well as patient frailty, to ensure a focus on efficient practice does not ignore the spectrum of diverse surgical practice across the NHS. Our results demonstrate even when the targeted 85% touchtime utilisation is met, it does not always correlate with the target of >75% total surgical cutting time as a proportion of total touchtime (Table 3).

Theatre turnover time constitutes a recognised segment within a theatre list, capable of inducing meaningful delays. Goldhaber et al. illustrated that the subdivision of theatre turnover over time into discrete tasks, coupled with tracking the duration of each task, resulted in a heightened understanding of delay causes, increased staff awareness regarding delays, and enhanced accountability for operational efficiency [8]. This paper emphasised that the use of a single measure of theatre utilisation may hide causes of delay. Ang et al. demonstrated the sizeable delays that can occur due to operative turnaround time and highlighted the potential savings to the NHS of £347,327.00 per theatre per year that improved efficiency could yield [9]. It is clear from such work that more efficient surgical practice is not only vital for an elective recovery in the NHS, but it should contribute to substantial cost savings as well.

We acknowledge the limitations of our study, including only reviewing orthopaedic operating lists at one MTC and one elective NHS hospital in North-West London. This may not be representative of the rest of England or the UK due to local factors. The total touchtime and total surgical cutting time may be skewed due to certain factors distinctive to orthopaedic surgery, including frailty of the patient population, comorbidity of the patient population, and choice of anaesthetic (spinal anaesthetic/regional nerve blocks/general anaesthetic) compared with other surgical specialities. Differences in practices based on speciality and acute versus elective may affect what is considered efficient practice, thus requiring further evaluation to ensure that national standards are valid and fair. Human factors limiting this study include the reliance on the surgical scrub team correctly documenting each of the time points correctly on the electronic patient record system.

There are inherent factors that differ between elective and emergency care operating, which will contribute to the differences between the results for the MTC and the EH, including patient population factors (acute patient physiology, urgency of surgery, frailty, opportunity for preoperative assessment) and hospital factors (availability of specialist services, operating room resources). However, we have ensured to include both within the study to ensure our results reflected differences across both types of practice.

We have also used capped touchtime utilisation for this study. In the case of overrunning lists or a list in which the start and finish times are slightly adjusted by the team, the capped touchtime utilisation would be lower than the uncapped touchtime utilisation with a higher potential gains statistic [10]. This implies more work could be done by the team, which is inaccurate as they have done more work without this being accounted for. It is important to be aware of this when formulating plans to increase efficiency.

The authors acknowledge that GRIFT's use of touchtime utilisation to measure theatre efficiency might be driven by its relative ease of calculation, broad applicability across different hospitals, trauma and elective care settings, and the complexity of patient care. This enables a surface-level evaluation of theatre efficiency.

As of April 2024, there are 7.57 million cases, and approximately 6.33 million patients on NHS waiting lists for consultant-led elective care [11]. National policy is critical to ensure maximum utilisation of surgical facilities to address this substantial backlog. It is imperative that the national directives aimed at mitigating the backlog in surgical care undergo validation, thereby enabling clinicians, clinical leaders, and managers to implement a performance-targeted strategy.

## Conclusions

Touchtime utilisation can be used as a crude measure of productivity and correlates to surgical cutting time; however, the differences between the two can be so great that in isolation it offers virtually no measure of operative/cutting time efficiency. Using touchtime utilisation in isolation fails to reliably indicate areas of efficiency and those requiring improvement. These factors can misrepresent inefficient practices in managing theatre time, potentially leading to increased costs and longer waiting lists. Based on the results of this study, we would suggest that to have a national policy focused on efficient surgical practice, there is a need to complement touchtime measures with surgical cutting time so that efficiency in practice is more clearly demonstrated and monitored, although further study and validation of such measures are warranted.
